# The evolution of comparative genomics

**DOI:** 10.1002/mgg3.112

**Published:** 2014-09-05

**Authors:** James C Mullikin

**Affiliations:** Comparative Genomics Analysis Unit, National Human Genome Research Institute, National Institutes of HealthBethesda, Maryland, 20892

## Introduction

The field of comparative genomics arose hand-in-hand with the ability to generate genomic sequence data. The first computer algorithms to compare amino acid sequences were developed over forty years ago (Fitch 1966; Needleman and Wunsch 1970)and improved upon as nucleic acid sequencing advanced (Sanger et al. 1977)with the application of improved statistical methods to the growing database of DNA sequence (Smith and Waterman 1981). This trend of exponentially increasing volumes of protein and DNA sequences has inspired a variety of algorithmic methods for DNA sequence comparison depending on the goal of a given investigation. BLAST (Altschul et al. 1990) is probably the best known of the alignment tools used today, but many others have been developed for specific comparative genomics studies, a few of which I will expand upon below.

## Interspecies Comparative Genomics

As the human genome sequencing projects raced toward high-quality draft assemblies (Lander et al. 2001; Venter et al. 2001), the mouse genome sequencing project (Mouse Genome Sequencing Consortium 2002) was in high gear as well, because it was already understood that the power of comparing the genomes of these two species would be immensely informative for both understanding the human genome and for understanding the genome of one of the most studied laboratory animal species. One of the big mysteries of the human genome was: if the gene coding regions only make up about 1.5% of the human genomic DNA sequence and 50% is repetitive sequence, how much of remainder is functionally important as defined by excess sequence similarity between these two species? The answer required accurate alignment of the two genomes, and existing software algorithms at the time were either not sensitive enough or would have taken excessive compute time. To address this new challenge, a new software package, called BLASTZ was created. As the name of this specially developed program implies, BLASTZ (Schwartz et al. 2003) is based on the strategies of BLAST (Altschul et al. 1990), but optimized for whole genome alignments of diverged species. One optimization relied on having relatively high contiguity sequences, and even though the mouse and human genomes were called draft genomes, they were both of high enough quality to allow the program to assume that the matching regions occur in the same order and orientation in both sequences. The other optimization was to use a different scoring matrix for nucleotide substitutions and sequence gaps. These primary optimizations along with many other improved methodological approaches, all nicely detailed in BLASTZ manuscript, allowed these two genomes to be aligned in 481 central processing unit (CPU) days, and with 1024 CPUs available to the group, the wall clock time was less than a day. This essential comparative genomics step then allowed many others to start interpreting the results, with one being a statistical estimate of functionally constrained fraction of the human genome relative to the mouse genome, which when analyzed in 50 base-pair windows across the genomes totaled 5%, or 140 Mb of human genomic DNA.

This number, 5%, was tantalizing in that we knew there were many more functionally important regions in the genome at the same level as coding sequence (CDS), but the locations of these regions were not as rigorously defined as CDSs. Thus in 2003, the **ENC**yclopedia **O**f **D**NA **E**lements (ENCODE) was launched to develop a variety of methods to “identify and precisely locate all of the protein-coding genes, non-protein coding genes and other sequence-based functional elements contained in the human DNA sequence. (http://www.genome.gov/10506706)” One of the key approaches was to use multispecies comparative genomics to improve the sensitivity and specificity of these elements. In the pilot phase of ENCODE Project Consortium (2007), 30 Mb (1%) of the human genome divided across 44 regions were selected for intense functional analyses including multispecies sequencing of orthologous regions in 28 other species. Total sequence across all the species and orthologous regions was 546 Mb, and represented a new challenge for comparative genomic analyses. This time three different software packages (Brudno et al. 2003; Blanchette et al. 2004; Bray and Pachter 2004) were developed for alignment of the multispecies genomic sequences because the subsequent detection of the evolutionarily constrained regions was quite sensitive to the final alignments produced. Now with more species compared the resolution of the constrained regions improved to a median length of 19 bases and a minimum size of 8 bases, and overall, the total fraction of the human genome under evolutionary mammalian constraint remained at 5%, a testament to power of the original human-mouse comparative analysis result. However, the overlap of CDS (32%), UTRs (8%), and other ENCODE detected functional elements (20%) still left 40% of the genome identified as important using comparative genomics but with unknown function.

**Figure 1:**
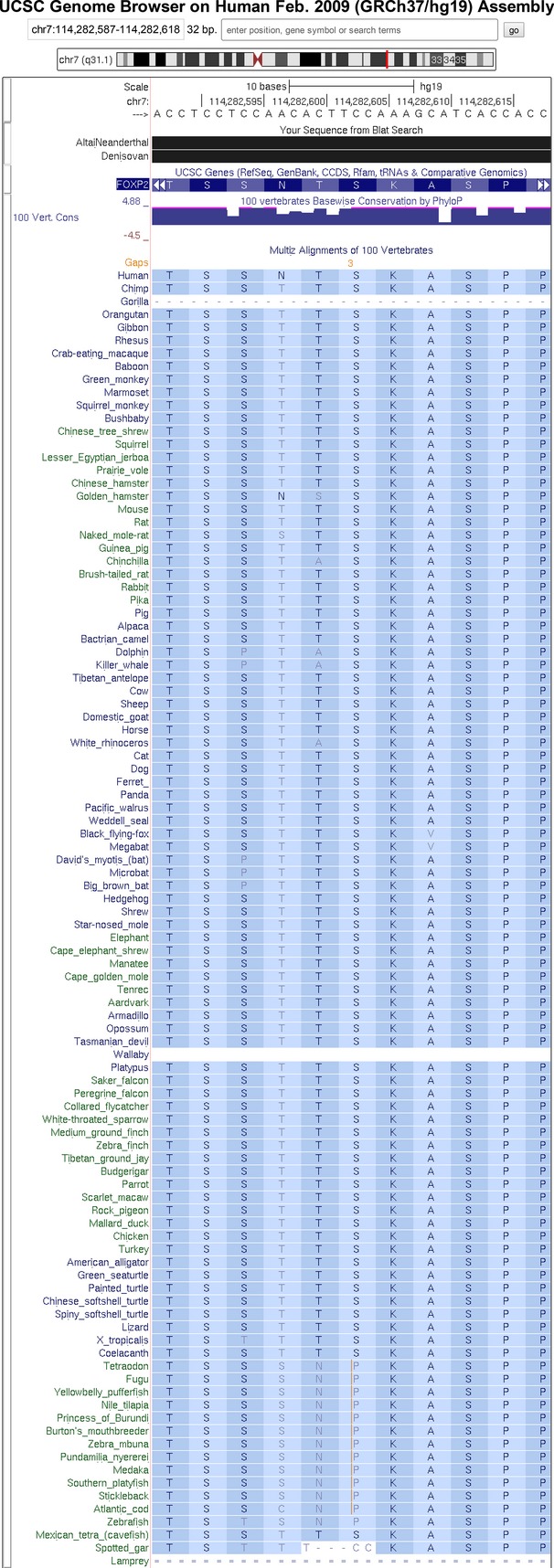
This UCSC genome browser image of a 32 base-wide window of the FOXP2 gene overlaps with one of the two human “speech” amino acid adaptation alleles (Enard et al. 2002), from a threonine as the ancestral allele to an asparagine in the human genome. Note that most of the alleles in the fourth column of the 100-way multispecies alignment is threonine, thus highly conserved. However, along with human, the Altai Neanderthal and the Denisovan genomes agree at the nucleotide level, as show with the solid black bars from a UCSC blat alignment of the orthologous sequence from these hominins’ genomes, indicating that this change happened after the split of hominins from the human–chimpanzee common ancestor and was fixed before the split of the human–neanderthal–denisovan common ancestor.

With the main phase of the ENCODE project now completed (Bernstein et al. 2012), we have a much more complete map of functional elements across the entire human genome. For this more recent genome-wide study, interspecies comparative genomics methods were applied to whole genomes of 29 mammals selected to maximize divergence across the four major mammalian clades (Lindblad-Toh et al. 2011). This resulted in a total effective branch length of 4.5 substitutions per site which, for example, translates into an incredibly infinitesimal probability of <10^−25^ that a window of 12 nucleotides that are not under purifying selection will remain fixed across all 29 species. Today, the most resent compilation of genome-wide comparative genomic analyses includes 100 vertebrate species (http://www.genome.ucsc.edu), see Figure 1, and provides a tremendous resource to the community in interpreting the genome from an evolutionary foundation which was built upon decades of improvements in sequencing, computational, and statistical methods. Looking into the near future, the Genome 10K Project (https://genome10k.soe.ucsc.edu/) is coordinating the collection of samples from over 10,000 vertebrate species specifically designated for whole-genome sequencing to better understand vertebrate evolution (Genome 2009).

## Intraspecies Comparative Genomics

In contrast to multispecies comparative genomics, intraspecies comparative genomics is used to find the variation across individuals of a given species. The first systematic effort to find large numbers of single-nucleotide polymorphisms (SNPs) in the human genome was through The SNP Consortium, which started generating data specifically for this effort in 1999 and completed in 2001. The original goal was to find at least 300,000 SNPs to give researchers landmarks across the genome to use for genetic association and linkage testing. This effort proved much more effective than originally planned, largely due to the acceleration of the Human Genome Project (Lander et al. 2001) during that time, with a final collection of over 1.4 million SNPs (Sachidanandam et al. 2001). The initial approach to discover SNPs did not require having the reference genome, because at the start of the project it was only 20% finished. Thus an approach called reduced representation shotgun sequencing (Altshuler et al. 2000) was developed, and proved to be an effective way to get enough sequencing reads to overlap to allow detection of variation from only a few hundreds of thousands of Sanger sequencing reads, instead of the then cost-prohibitive tens of millions of reads without this approach. However, by 2001, the draft human genome was nearly complete and random shotgun sequence from selected human genomic DNA samples proved to be much more cost effective. With these initial 1.4 million SNPs available, the focus turned toward understanding and mapping the haplotype structure of the human genome, however, other, more focused efforts, were indicating that many more SNPs were required to more completely resolve the haplotype map of the human genome (Mullikin et al. 2000). Thus, at the start of the human haplotype map project (HapMap) in 2003, focus continued on SNP discovery using random whole-genome shotgun sequences from individuals of European, Asian, and African ancestry and all compared to the improving human reference sequence. To map these Sanger reads, with lengths of 400–800 nucleotides in length, I developed and used the ssahaSNP algorithm (Ning et al. 2001) on the rapidly increasing number of reads generated by the genome sequencing centers. The optimizations of this algorithm used assumptions that the sequence of a given read would match with very few differences, so that the reference sequence could be indexed very efficiently in a large memory LINUX computer (over 12 gigabytes of random access memory) and the process of alignment became a memory lookup operation followed by a fast local alignment algorithm, making the speed of aligning a read to a reference genome essentially independent of the genome size. Even with the computers available in 2001, alignments raced along at 200 reads per second, which was three to four orders of magnitude faster than the version BLAST available then. The HapMap project contributed another six million SNPs to dbSNP, bringing the total in dbSNP to 9.2 million SNPs in October of 2005 (International HapMap Consortium 2005). With this SNP set available and a high-throughput genotype technology from Perlegen, phase II of HapMap proceeded quite quickly, culminating with a combined total of 3.1 million SNPs genotyped across 270 individuals from four geographically diverse populations (International HapMap Consortium 2007). The end result of these efforts and the continued improvements in genotyping technologies which utilized optimal subsets of SNPs based on the haplotype structures of the human genome populations enabled the huge expansion of genome-wide association studies (GWAS) which was reported in an earlier commentary in this journal (Adeyemo and Rotimi 2014). Furthermore, SNP discovery has been applied to hundreds of other species; see dbSNP (http://www.ncbi.nlm.nih.gov/SNP/index.html) for summaries of SNPs available across the kingdoms of life.

## Comparative Genomics Insights into Hominin Evolution

Paleoanthropology over the last 150 years has built a tree of hominin evolution based on fossils that date back over the last 4–5 million years. Some recent and well preserved fossils of now extinct hominins dating back 30–100,000 years ago have been shown to contain enough endogenous DNA to allow us to sequence their genomes, and by comparing these archaic genomes to modern humans, gain new insights into human evolution. The first attempt to extract and sequence DNA from a Neanderthal bone targeted the hyper-variable region of the mitochondria (Krings et al. 1997). Using 13 overlapping PCR primer-pair amplification products, Dr. Pääbo's group was able to generate 379 bases of contiguous consensus sequence and compared this to modern human sequence and chimpanzee sequence of the same mitochondrial region, thus started the era of paleogenomics. As the sequencing technologies and methods advanced, first with the arrival of the 454 sequencing instrument and later with the Solexa, now Illumina, massively parallel sequencing instrument, sequencing the entire genome of the Neanderthal was completed (Green et al. 2010). Subsequently, with the discovery of a very well preserved Neanderthal toe bone from the Denisova cave in Altai mountains along with advancements in archaic DNA extraction methods and sequencer throughput, a new and much improved Neanderthal genome was completed (Prufer et al. 2014).

One of the primary questions we hoped to find an answer to from the genomes of our closest archaic ancestors: is there any evidence, or not, of interbreeding when humans encountered Neanderthals as they left Africa and entered the domain that Neanderthals had occupied for the previous 400,000 years? The method to detect this required, in addition to the Neanderthal genome, whole genome sequences of modern humans from a variety of ancestral population locations. In the earlier work, these sequences were ascertained from five individuals, one San from Southern Africa, one Yoruba from West Africa, one Papua New Guinean, one Han Chinese, and one French from Western Europe. Each was sequenced to four- to sixfold coverage on the Illumina GAII platform. To avoid biased results by comparing these sequences to the reference human genome, which is a mosaic assembly of a few individuals of various ancestral population locations, the sequences were compared to the chimpanzee genome since the common ancestor to humans and chimpanzees predates the common ancestor to humans and Neanderthals, and the chimpanzee genome sequence is similar enough to the human and Neanderthal genomes sequence to allow robust alignments. If there had been interbreeding of Neanderthals with humans that left Africa between 30–80,000 years ago, and if their offspring remained in a geographical area, e.g., Europe, since that time, comparing pairs of modern human genomes, say a European and a Papua New Guinean, then one looks at all positions where there are differences between these present-day humans and count how many times the Neanderthal genome agrees with one versus the other. If the Neanderthal allele agreeing counts are statistically higher for the individual of European ancestry versus the individual of Papua New Guinea ancestry, that would show evidence of greater Neanderthal contribution to the European than to the Papua New Guinean. Putting this in a statistical framework, the “D” statistic developed for this very analysis, was able to determine evidence of interbreeding that occurred early in the migration of humans leaving Africa, about 50–80,000 years ago, since all three out-of-Africa ancestry individuals contained approximately the same skew of more Neanderthal alleles when compared to the Southern African or West African individuals. A similar analysis was repeated with the sequence of the Neanderthal individual from the Altai Mountains, along with an increased number of 25 present-day human genomes and the evidence for interbreeding remained, along with additional gene flow signatures, see figure 8 in (Prufer et al. 2014), which also incorporated a newly discovered hominin from the same Denisova cave (Krause et al. 2010; Meyer et al. 2012).

## Summary

**Figure 2:**
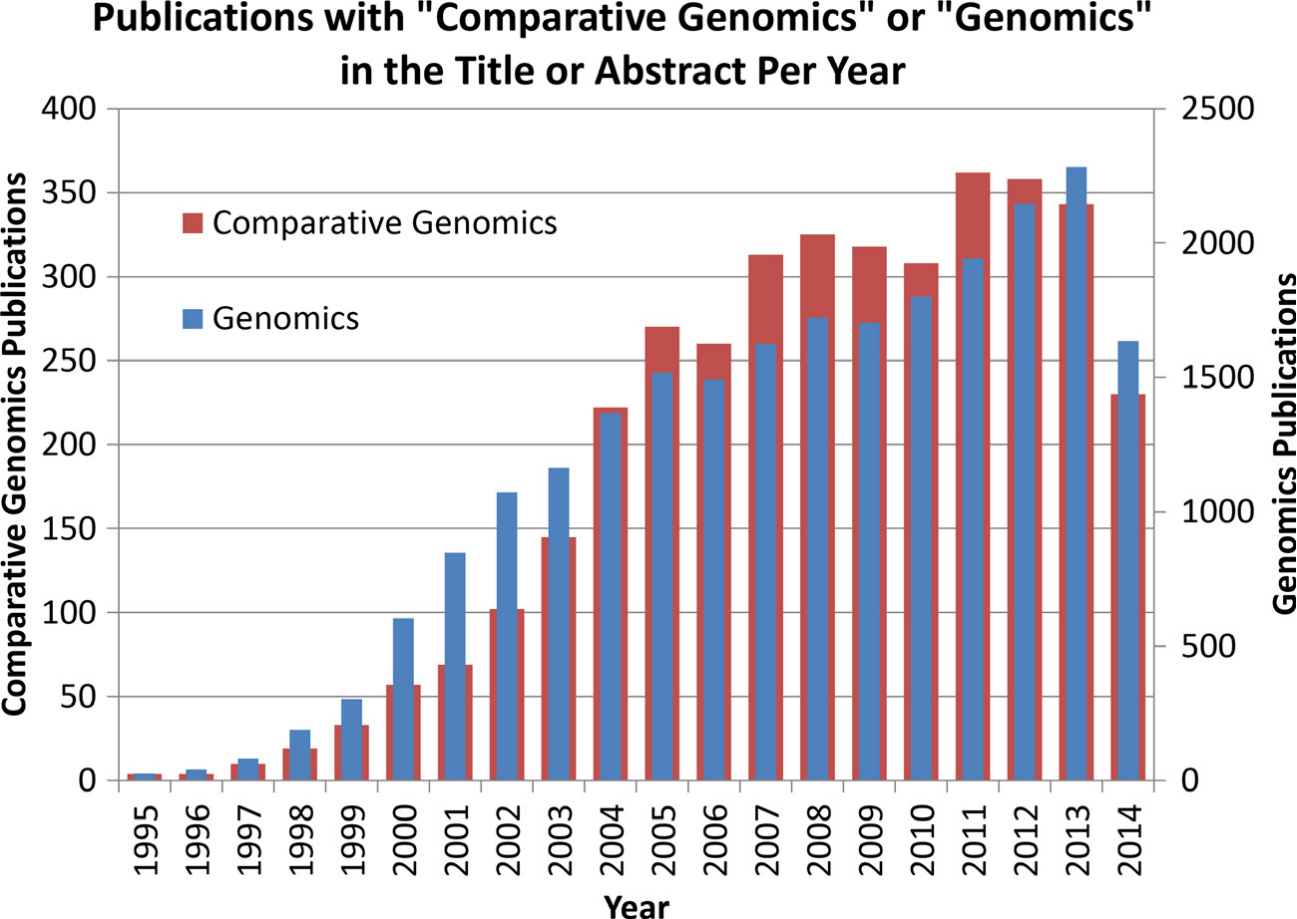
A PubMed search of publications with the exact combination and order of the words “comparative genomics” in the title or abstract identifies 3752 articles. This chart shows the growth of publications in this field year-by-year, and for comparison includes the same search for “genomics.”

In this commentary I have only highlighted a few dimensions that comparative genomics has reached into. Looking at a PubMed search of publications with the exact combination and order of the words “comparative genomics” in the title or abstract identifies 3752 articles as of the date of this writing. The chart in Figure 2 shows the growth of this field, which at first lagged in growth relative to the same search for “genomics,” but overall tracks this more general field of research. Other dimensions of comparative genomics, beyond the three areas I touched on above, include intraindividual comparative genomics (Cheng et al. 2012; Biesecker and Spinner 2013; Watson et al. 2013), human microbiome comparative genomics (Human Microbiome Project Consortium 2012) and how comparative genomics can shed light on a multidrug-resistant bacteria spread through a hospital (Snitkin et al. 2012). Clearly, as the field of genomics continues to expand, comparative genomics will always be an essential and central enabling component to help us discover and better understand the complexities, intricacies, and interrelatedness of the genomics of life.
